# Afterhyperpolarization potential modulated by local [K^+^]_o_ in K^+^ diffusion-restricted extracellular space in the central clock of suprachiasmatic nucleus

**DOI:** 10.1016/j.bj.2022.07.005

**Published:** 2022-07-19

**Authors:** Jyh-Jeen Yang, Rong-Chi Huang

**Affiliations:** a Department of Physiology and Pharmacology, College of Medicine, Chang Gung University, Taoyuan, Taiwan; b Healthy Aging Research Center, Chang Gung University, Taoyuan, Taiwan; c Neuroscience Research Center, Chang Gung Memorial Hospital at Linkou, Taoyuan, Taiwan

**Keywords:** Action potential, Afterhyperpolarization potential, Extracellular space, Na^+^/K^+^-ATPase, Resting membrane potential, Suprachiasmatic nucleus

## Abstract

**Background:**

Intercellular coupling is essential for the suprachiasmatic nucleus (SCN) to serve as a coherent central clock. Synaptic release of neurotransmitters and neuropeptides is critical for synchronizing SCN neurons. However, intercellular coupling via non-synaptic mechanisms has also been demonstrated. In particular, the abundant perikaryal appositions with morphological specializations in the narrow extracellular space (ECS) may hinder molecular diffusion to allow for ion accumulation or depletion.

**Methods:**

The SCN neurons were recorded in the whole-cell current-clamp mode, with pipette filled with high (26 mM)-Na^+^ or low (6 mM)-Na^+^ solution.

**Results:**

Cells recorded with high-Na^+^ pipette solution could fire spontaneous action potentials (AP) with peak AHP more negative than the calculated value of K^+^ equilibrium potential (E_K_) and with peak AP more positive than calculated E_Na_. Cells recorded with low-Na^+^ pipette solution could also have peak AHP more negative than calculated E_K_. In contrast, the resting membrane potential (RMP) was always less negative to calculated E_K_. The distribution and the averaged amplitude of peak AHP, peak AP, or RMP was similar between cells recorded with high-Na^+^ and low-Na^+^ solution pipette. In a number of cells, the peak AHP could increase from more positive to become more negative than calculated E_K_ spontaneously or after treatments to hyperpolarize the RMP. TTX blocked the Na^+^ -dependent APs and tetraethylammonium (TEA), but not Ba^2+^ or Cd^2+^, markedly reduced the peak AHP. Perforated-patch cells could also but rarely fire APs with peak AHP more negative than calculated E_K_.

**Conclusion:**

The result of peak AHP negative to calculated E_K_ indicates that local [K^+^]_o_ sensed by the TEA-sensitive AHP K^+^ channels must be lower than bulk [K^+^]_o_, most likely due to K^+^ clearance from K^+^ diffusion-restricted ECS by the Na^+^/K^+^-ATPase. The K^+^ diffusion-restricted ECS may allow for K^+^-mediated ionic interactions among neurons to regulate SCN excitability.

**At a glance of commentary**

**Scientific background on the subject**

Intercellular coupling is essential for the SCN to serve as a coherent central clock. Whereas synaptic release of neurotransmitters/neuropeptides is critical for synchronizing SCN neurons, non-synaptic communication is also present. In particular, the SCN is tightly packed with small neurons and has abundant perikaryal appositions with narrow extracellular space.

**What this study adds to the field**

The rat SCN neurons can fire spontaneous action potentials with peak AHP more negative than calculated E_K_. The result indicates a lower-than-bulk local [K^+^]o sensed by the AHP K^+^ channels in the K^+^ diffusion-restricted extracellular space, which may allow for K^+^-mediated ionic interactions among neurons to regulate SCN excitability.

The suprachiasmatic nucleus (SCN) in the hypothalamus is the central clock that coordinates peripheral clocks to control circadian rhythms in mammals [1]. The SCN neurons exhibit a circadian rhythm in spontaneous firing rate both in intact animals [2] and in isolated hypothalamic slices [[3], [4], [5]]. The autonomous oscillation in the circadian clock arises from a transcriptional translational feedback loop leading to ∼24 h oscillation of clock genes and proteins within individual SCN neurons [6,7]. Indeed, most individual SCN neurons in dissociation still exhibit circadian variation in the spontaneous firing rate, albeit with various periods and phases [[8], [9], [10]]. The scattered periods and phases become narrower in SCN explants [8] and narrowest in the circadian period of locomotor activity [8,9]. Together, the results indicate that the SCN contains cell-autonomous circadian oscillators that are synchronized to act as a coherent oscillator.

Intercellular coupling of SCN neurons is critically dependent on chemical communication via the release of neurotransmitters and neuropeptides, particularly GABA and vasoactive intestinal peptide (for review, see Ref. [11]). However, non-synaptic communication must be present to account for the functional circadian clocks in the developing SCN before the occurrence of significant synaptic connections [[12], [13], [14]]. In the rat SCN, neuronal synchronization is known to occur in the absence of Ca^2+^-dependent synaptic transmission [15]. The SCN is tightly packed with small neurons and has abundant perikaryal appositions [16], which may favor non-synaptic communications such as electrotonic coupling via gap junctions [[17], [18], [19]] and ionic interactions (see Ref. [20]). In particular, the abundant perikaryal appositions with morphological specializations in the narrow extracellular space (ECS) could increase the tortuosity to effectively slow molecular diffusion, allowing ion accumulation or depletion to occur in the diffusion-restricted ECS.

In this study, we used patch-clamp recordings to investigate the spontaneous AP in neurons from reduced, acute rat SCN slices. We showed that the SCN neurons could fire spontaneous APs with large peak afterhyperpolarization potential (AHP) more negative than the calculated value of K^+^ equilibrium potential (E_K_) in whole-cell and even in perforated-patch recordings. In contrast, the resting membrane potential (RMP) was always less negative than calculated E_K_. The result indicates that local [K^+^]_o_ in the ECS sensed by the AHP K^+^ channels must be lower than bulk [K^+^]_o_, most likely due to K^+^ clearance from the K^+^ diffusion-restricted ECS by the Na^+^/K^+^-ATPase. The ability of local [K^+^]_o_ to regulate peak AHP amplitude may allow for the K^+^ diffusion-restricted ECS to mediate ionic interactions among neurons to regulate SCN excitability.

## Material and Methods

### Hypothalamic brain slices and reduced SCN preparations

All experiments were carried out according to procedures approved by the Institutional Animal Care and Use Committee of Chang Gung University (IACUC Approval No.: CGU109-110). Sprague–Dawley rats (18–24 days old) were kept in a temperature-controlled room under a 12:12 light:dark cycle (light on 0700–1900 h). Lights-on was designated Zeitgeber time (ZT) 0. For daytime and nighttime recordings, the animal was killed at ZT 2 and ZT 10, respectively. Hypothalamic brain slices and reduced SCN preparations were made as described previously [21,22]. An animal of either sex was carefully restrained by hand to reduce stress and killed by decapitation using a small rodent guillotine without anaesthesia, and the brain was put in an ice-cold artificial cerebrospinal fluid (ACSF) prebubbled with 95% O_2_–5% CO_2_. The ACSF contained (in mM): 125 NaCl, 3.5 KCl, 2 CaCl_2_, 1.5 MgCl_2_, 26 NaHCO_3_, 1.2 NaH_2_PO_4_, 10 glucose. A coronal slice (200–300 μm) containing the SCN and the optic chiasm was cut with a DSK microslicer DTK-1000 (Ted Pella, Redding, CA, USA), and was then incubated at room temperature (22–25 °C) in the incubation solution, which contained (in mM): 140 NaCl, 3.5 KCl, 2 CaCl_2_, 1.5 MgCl_2_, 10 glucose, 10 HEPES, pH 7.4, bubbled with 100% O_2_.

For electrical recordings, a reduced SCN preparation was obtained by excising a small piece of tissue (circa one-ninth the size of SCN) from the medial SCN using a fine needle (Cat no. 26002-10, Fine Science Tools, Foster City, CA, USA), followed by further trimming down to 4–10 smaller pieces with a short strip of razor blade. The reduced preparation (containing tens to hundreds of cells, see Fig. 1 of ref. [22]) was then transferred to a coverslip precoated with poly-d-lysine (Sigma–Aldrich, St Louis, MO, USA) in a recording chamber for recording. The SCN neurons of the reduced preparation could be identified visually with an inverted microscope (Olympus IX70, Japan). The preparation thus obtained allows rapid application of drugs [23] and has been used to demonstrate diurnal rhythms in both spontaneous firing and Na^+^/K^+^-ATPase activity [24].

**Fig. 1:**
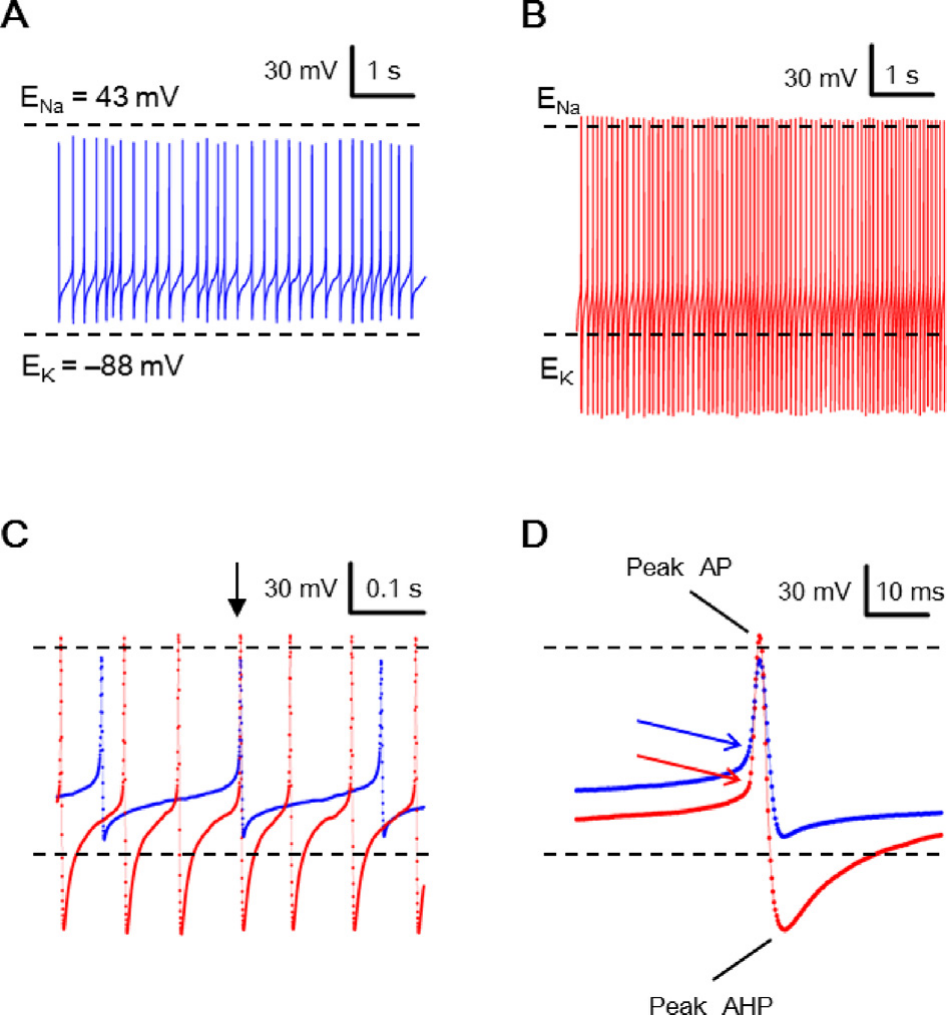
Spontaneous action potentials (APs) recorded with high-Na^+^ pipette solution. (A) A representative SCN neuron to show spontaneous APs with peak AP and peak AHP bounded between calculated E_Na_ and E_K_. (B) A representative SCN neuron to show spontaneous APs with peak AP positive to calculated E_Na_ and peak AHP negative to calculated E_K_. (C) APs from (A) (blue trace) and from (B) (red trace) were superimposed to compare their peak AHP and interspike potential. Arrow marks the two APs to be compared in (D). (D) APs with expanded time course to show the AP threshold (marked by arrows).

### Electrical recordings

Electrical recordings were carried out as described previously [25]. The reduced SCN preparation was perfused with bath solution containing (in mM): 140 NaCl, 3.5 KCl, 2 CaCl_2_, 1.5 MgCl_2_, 10 glucose, 10 HEPES, pH adjusted to 7.4 with NaOH. The perfusion rate was set at 0.6 ml/min and solution change was completed in ∼1 s judging from the measurement of junction potential. The patch solution contained (in mM): 20 NaCl (high-Na^+^) or 20 KCl (low-Na^+^), 1 CaCl_2_, 2 MgCl_2_, 110 K-gluconate, 11 EGTA, 10 HEPES, 3 Na-ATP, 0.3 Na-GTP, pH adjusted to 7.3 with KOH. The measured liquid junction potential was −12 mV [26] and was corrected for in the presentation of data obtained with whole-cell and perforated-patch recordings. Pipette resistance was 4–6 MΩ. For perforated-patch recordings, the patch pipette also included nystatin (Sigma–Aldrich, St Louis, MO, USA) at a final concentration of 250 μg/ml prepared from a stock solution (25 mg/ml DMSO). All recordings were made with Axopatch 200B amplifier (Axon Instruments, Foster City, CA, USA) at room temperature (22–25 °C). Membrane potentials were recorded using the whole-cell and perforated-patch recording techniques. The signal was low-pass filtered at 1–5 KHz and digitized on-line at 2–10 KHz via a 12-bit A/D digitizing board (DT2821F-DI, Data Translation, Marboro, MA, USA) with a custom-made program written in the C Language. Data were analyzed and plotted with custom-made programs written in Visual Basic 6.0 and the commercial softwares GraphPad PRISM (GraphPad Software, San Diego, CA, USA) and Stata (StataCorp LLC, College Station, Texas, USA). Data are given as means ± SEM and were analyzed with Student's *t*-test and paired *t*-test. Two-sample Kolmogorov–Smirnov test for equality of distribution functions was used to compare the data distributions between cells recorded with high-Na^+^ and low-Na^+^ pipette solutions [Fig. 3].

**Fig. 2:**
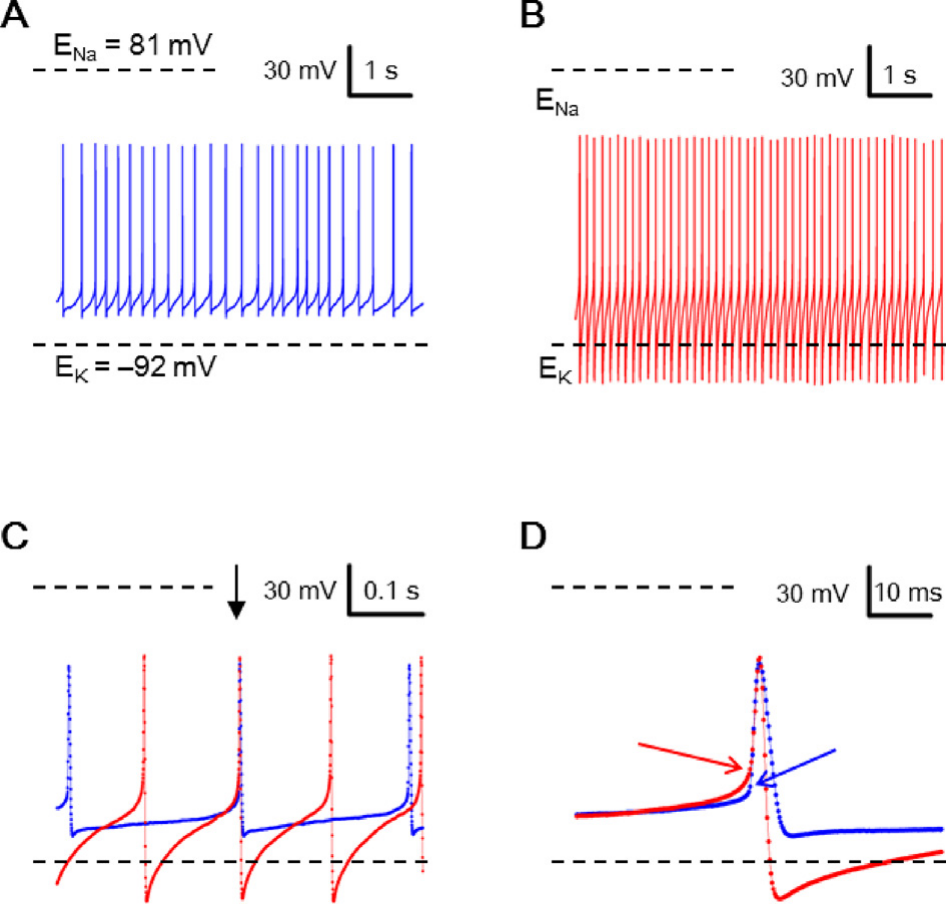
Spontaneous action potentials (APs) recorded with low-Na^+^ pipette solution. (A) A representative SCN neuron firing spontaneous APs with peak AP and peak AHP bounded between calculated E_Na_ and E_K_. (B) A representative SCN neuron firing spontaneous APs with peak AHP negative to calculated E_K_. (C) APs from (A) (blue trace) and from (B) (red trace) were superimposed to compare their peak AHP and interspike potential. Arrow marks the two APs to be compared in (D). (D) APs with expanded time course to show the AP threshold (marked by arrows).

**Fig. 3:**
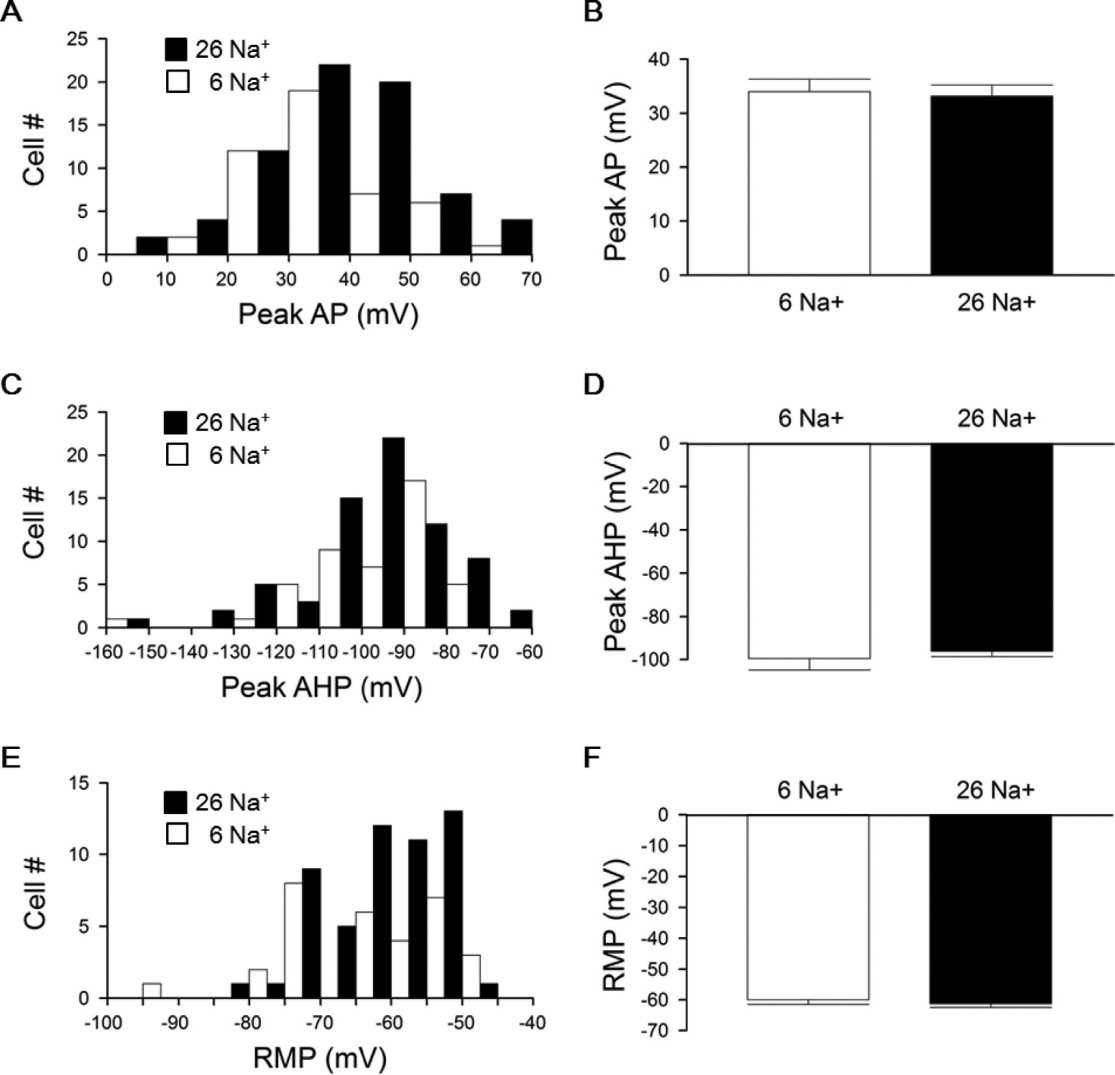
Distribution and averaged amplitude of peak AP, peak AHP, and RMP between cells recorded with high-Na^+^ (filled bars) and low-Na^+^ (open bars) pipette solution. Note the similar distribution of peak AP (10 mV bin size; A), peak AHP (10 mV bin size; C), and RMP (5 mV bin size; E) between the two groups. There is also no difference between high-Na^+^ and low-Na^+^ in the average amplitude of peak AP (B), peak AHP (D), and RMP (F).

### Drugs

The stock solution of TTX (0.3 mM in acetic acid) was stored at −20 °C and was diluted 1000 times to reach desired final concentrations. TTX was from Tocris Cookson Inc. (Ellisville, MO, USA). Carbachol, Cd^2+^, Ba^2+^ and TEA were purchased from Sigma–Aldrich.

## Results

### SCN neurons generate APs with peak AHP negative to calculated E_K_

The spontaneous firing of rat SCN neurons was investigated using the whole-cell recording technique, with pipette filled with high-Na^+^ (26 mM Na^+^/110 mM K^+^) or low-Na^+^ (6 mM Na^+^/130 mM K^+^) solution. For the experiments, the SCN neurons were recorded in the current-clamp mode after breaking into the whole-cell configuration, and the membrane potential was recorded in 6-s epochs. Only cells with the peak-to-peak amplitude of the AP larger than 100 mV were included in the analysis.

Fig. 1 shows the results from two representative cells in the first minute after breaking into the cells with high-Na^+^ pipette solution. Fig. 1A shows an epoch of 6 s membrane voltage recorded from a cell firing spontaneous APs with expected magnitudes of peak AP and AHP that were bounded between the calculated values of Na^+^ equilibrium potential (E_Na_) and E_K_. Fig. 1B shows another cell that fired spontaneous APs with large amplitudes of peak AP and peak AHP beyond calculated E_Na_ and E_K_, respectively. Comparison of the APs reveals that the faster firing neuron had a faster depolarizing rate of interspike potential, apparently as a result of much larger peak AHP [Fig. 1C]. Incidentally, the AP threshold (marked by arrows) is also lower for the faster firing neuron (red) than the slower one (blue) [Fig. 1D]. For a total of 72 cells with high-Na^+^ pipette solution, 27 cells (37%) have peak AP positive to calculated E_Na_ (+43 mV), 49 cells (68%) peak AHP negative to calculated E_K_ (**–**88 mV), and 18 (24%) cells both peak AP and peak AHP more positive and negative than calculated E_Na_ and E_K_, respectively (see Fig. 3A and C; filled bars). In contrast, the resting membrane potential (RMP) was never (0 out of 53 cells) negative to calculated E_K_ (see Fig. 3E, filled bars).

The occurrence of peak AHP negative to calculated E_K_ indicates that local [K^+^]_o_ sensed by the TEA-sensitive AHP K^+^ channels [see Fig. 7D] must be lower than bulk [K^+^]_o_, which is clamped by the perfusion bath solution, most likely due to K^+^ depletion in the narrow ECS facing the membrane by the action of Na^+^/K^+^-ATPase (NKA). Likewise, the occurrence of peak AP positive to calculated E_Na_ indicates that submembrane local [Na^+^]_i_ sensed by the TTX-sensitive Na^+^ channels [see Fig. 7A] must be lower than the bulk [Na^+^]_i_, which is clamped by the high-Na^+^ pipette solution, most likely also due to extrusion of Na^+^ via NKA. Because NKA is activated by intracellular Na^+^ in the rat SCN neurons [27], the large peak AHP negative to calculated E_K_ and peak AP positive to calculated E_Na_ is arguably a consequence of heightened NKA pumping activity due to the use of high-Na^+^ pipette solution.

Therefore we also used low-Na^+^ pipette solution to investigate the spontaneous firing. Fig. 2 shows the result obtained from two representative cells, one with peak AP and peak AHP bounded between calculated E_Na_ and E_K_ [Fig. 2A] and the other with peak AHP negative to calculated E_K_ [Fig. 2B]. Comparison of the APs again indicates that the faster firing neuron had a faster depolarizing rate of interspike potential, as a result of larger AHP [Fig. 2C]. Interestingly, the AP threshold (marked by arrows) is actually higher for the faster firing neuron (red) than the slower one (blue) [Fig. 2D]. For a total of 47 cells with low-Na^+^ pipette solution, none (0%) have peak AP positive to calculated E_Na_ (+81 mV), but 24 cells (51%) have peak AHP negative to calculated E_K_ (**–**92 mV) (see Fig. 3A, C; open bars). Again, the RMP was never (0 out of 47 cells) negative to calculated E_K_ (see Fig. 3E, open bars).

Taken together, the results indicate that the SCN neurons could fire APs with peak AHP more negative than calculated E_K_ irrespective of high- or low-Na^+^ pipette solution, and with peak AP more positive than calculated E_Na_ only using high-Na^+^ solution. In contrast, RMP was always less negative than calculated E_K_. Fig. 3 compares the distribution and the averaged amplitude of peak AP [Fig. 3A and B], peak AHP [Fig. 3C and D], and RMP [Fig. 3E and F] from cells recorded with high-Na^+^ (filled bars) and low-Na^+^ (open bars) pipette solutions. The two-sample Kolmogorov–Smirnov test indicates similar distributions of peak AP (D = 0.1571; *p*-value = 0.481) [Fig. 3A], peak AHP (D = 0.1408; *p*-value = 0.623) [Fig. 3C], and RMP (D = 0.1481; *p*-value = 0.791) [Fig. 3E] between the two groups of cells. There is also no difference between high-Na^+^ and low-Na^+^ in the average amplitude of peak AP (33.2 ± 2.1 mV (n = 73) vs 34.0 ± 2.2 mV (n = 47); *p* > 0.05; Student's *t*-test) [Fig. 3B], peak AHP (−96.2 ± 2.5 mV (n = 73) vs −99.5 ± 5.3 mV (n = 47); *p* > 0.05; Student's *t*-test) [Fig. 3D], and RMP (−61.3 ± 1.2 mV (n = 53) vs −60.0 ± 1.5 mV (n = 31); *p* > 0.05; Student's *t*-test) [Fig. 3F]. It is interesting to note that despite the large difference in the values of calculated E_Na_ between high-Na^+^ (E_Na_ = +81 mV) and low-Na^+^ (E_Na_ = +43 mV) pipette solution, both the distribution [Fig. 3A] and averaged amplitude [Fig. 3B] of peak AP were similar. The result suggests that submembrane local [Na^+^]_i_ sensed by the TTX-sensitive Na^+^ channels was similar despite the large difference in bulk [Na^+^]_i_ clamped by the high- and low-Na^+^ pipette solution (see Discussion).

### Peak AHP negative to calculated E_K_ may arise later spontaneously

**Fig. 4:**
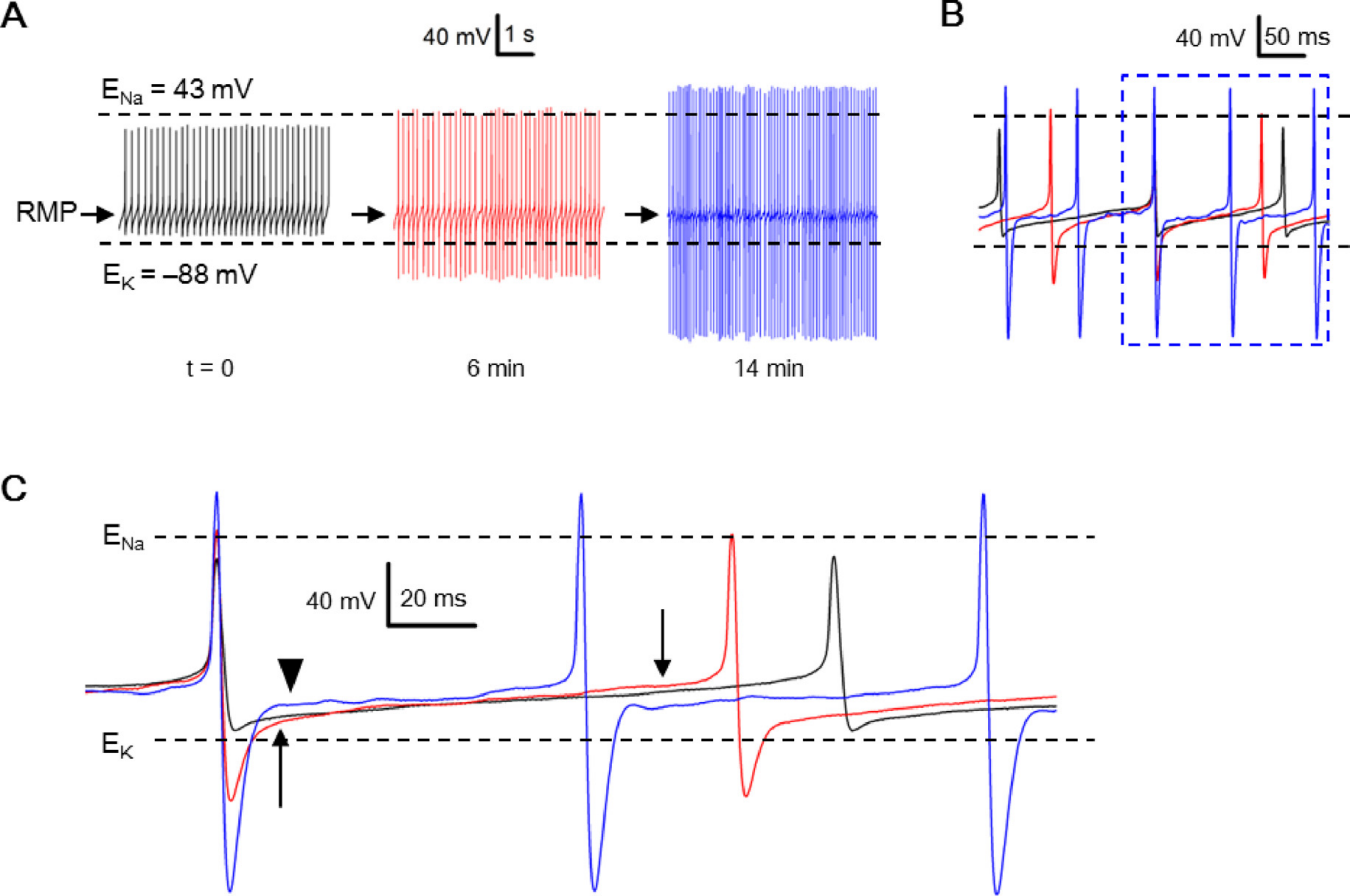
Spontaneous increase in the peak AHP amplitude recorded with high-Na^+^ pipette solution. (A) A representative SCN neuron showing its spontaneous firing recorded at t = 0 (left panel), 6 min (middle panel), and 14 min (right panel) after breaking into whole-cell recordings. Note the gradual increase in the peak-to-peak amplitude of the action potentials (APs). (B) Three selected stretch of voltage record, each from three different times (A), superimposed to compare their APs and interspike potentials. The broken lined box marks the area to be replotted in (C). (C) Expanded time course to compare the peak AHP and interspike potentials. Arrows mark the faster rate of depolarization for the interspike potential due to the larger peak AHP (red trace) compared to the smaller one (black trace). Arrowhead marks the larger depolarization rebound from an even larger peak AHP (blue trace).

The results presented so far were all obtained from the first minute of whole-cell recording. During the course of recording, however, a number of cells were observed to have their peak AHP spontaneously increased to become negative to calculated E_K_ [Fig. 4]. Fig. 4A shows a representative experiment to demonstrate a cell firing spontaneous APs with peak AP and peak AHP bounded between calculated E_Na_ (+43 mV) and E_K_ (**–**88 mV) at the beginning (t = 0) of whole-cell current-clamp recording with high-Na^+^ electrode solution (leftmost panel). The peak-to-peak amplitude became larger and larger as time passed, with peak AP/AHP increasing/decreasing from ∼+30/**–**80 mV (t = 0; leftmost panel), to ∼+45/**–**125 mV (t = 6 min; middle panel), and to ∼+70/**–**185 mV (t = 14 min; rightmost panel). For this particular cell, the peak AP became positive to calculated E_Na_ and peak AHP negative to calculated E_K_ at t = 6 min (middle panel) and both the peak AP and peak AHP became even larger at t = 14 min (rightmost panel). In sharp contrast, the approximate RMP remained virtually unchanged at **–**61 mV (marked by arrows).

Parallel to an increase in the magnitude of peak AHP, the firing rate also increased from 5.8 Hz (t = 0; left panel), to 7.2 Hz (t = 6 min; middle panel), and to 11.8 Hz (t = 14 min; right panel) [Fig. 4A], suggesting a correlation of peak AHP with the firing rate. This can be seen by superimposing a selected stretch of recordings with regular firing of a few APs to allow for better comparison of peak AHP with interspike interval as shown in Fig. 4B. The result indicates that the interspike interval decreases, or the firing rate increases, with larger peak AHP. Fig. 4C further expands the region enclosed by the broken lined box [Fig. 4B] to better illustrate the relation between peak AHP and interspike potential. The earlier increase in the firing rate, from t = 0 (black trace) to t = 6 min (red trace), is correlated with the increase in the slope (depolarizing rate) of interspike potential (marked by arrows) due to the larger peak AHP (red trace). The later increase in the firing rate, from time = 6 (red trace) to t = 14 min (blue trace), is apparently due to a larger depolarization (marked by arrowhead) rebound from a much larger peak AHP (blue trace). The result suggests that a larger peak AHP removes more resting inactivation of inward currents contributing to the interspike potential. For a total of 7 cells having their peak AHP increased spontaneously to become negative to calculated E_K_, the peak AHP amplitude changed from −82.3 ± 2.2 mV (n = 7) at t = 0 to −126.1 ± 12.4 mV (n = 7; *p* < 0.05; paired *t*-test) at t = 3–14 min and the spontaneous firing rate from 2.7 ± 0.9 Hz (n = 7) to 5.6 ± 1.6 Hz (n = 7; *p* < 0.05; paired *t*-test).

**Fig. 5:**
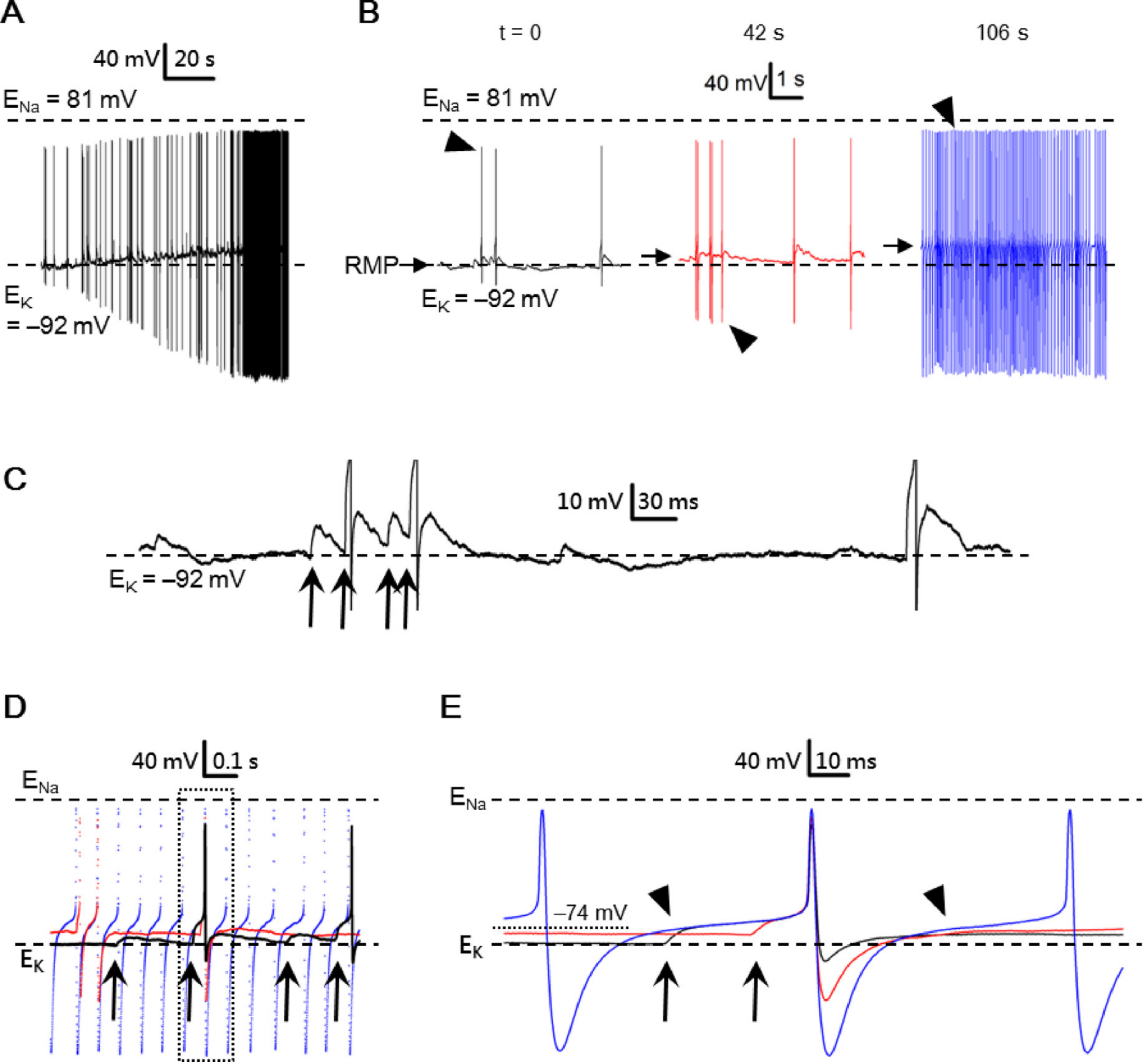
Spontaneous increase in the peak AHP amplitude recorded with low-Na^+^ pipette solution. (A) A representative SCN neuron showing rapid increase in the peak-to-peak amplitude of action potentials (APs). (B) Three 6-s epochs of membrane voltage from (A) recorded at t = 0 (left panel), 42 s (middle panel), and 106 s (right panel) after breaking into whole-cell recordings. Note the gradual increase in the RMP (marked by arrows). Arrowheads mark the AP to be compared in (D). (C) Expanded time course of truncated, enlarged 6-s voltage trace from (C; left panel) to show 4 consecutive synaptic inputs (marked by arrows), with two of them provoking APs. (D) Three selected stretch of voltage recordings from (B, marked by arrowheads) superimposed to compare their synaptic-driven and spontaneous APs. Arrows mark the synaptic potentials (black trace). Dotted box marks the region to be replotted in (E). (E) Expanded time course to compare the peak AHP and interspike potential. Arrows mark the synaptic potentials leading to APs. Arrowhead marks the interspike potential of spontaneous APs.

Fig. 5A shows a cell firing spontaneous APs with the peak-to-peak amplitude rapidly increased from a value of ∼160 mV at the beginning of whole-cell recordings to a surprisingly large value of ∼290 mV in less than 2 min; at the same time RMP gradually depolarized from **–**91 mV to **–**74 mV. Unlike the more gradually depolarizing RMP and larger peak AP and AHP, the firing rate increased from 0.5 Hz to 10 Hz more abruptly at a later time. Fig. 5B shows three 6-s epoch of membrane voltage recorded at t = 0 (leftmost panel), 42 s (middle panel), and 106 s (rightmost panel). Note the gradual increase in RMP (marked by arrows), from **–**91 mV (t = 0; leftmost panel), to **–**84 (t = 42 s; middle panel), and to **–**74 mV (t = 106 s; rightmost panel), as well as increase of firing rate from 0.5 Hz (leftmost panel), to 1.2 Hz (middle panel), and to 10 Hz (rightmost panel).

Note that, for this particular cell, on first breaking into the whole-cell recording mode (t = 0), RMP was close to the calculated E_K_ = **–**92 mV and the AP appeared to be driven by synaptic inputs (leftmost panel, Fig. 5B). Fig. 5C shows the enlarged and expanded voltage trace to illustrate a barrage of depolarizing synaptic potentials (marked by arrows) leading to APs. The cell began to fire spontaneous APs at a rate of 10 Hz when the approximate RMP had reached **–**74 mV (rightmost panel, Fig. 5B). For comparison, three selected stretch of recordings each from the three 6-s epoch (Fig. 5B, marked by arrowheads) are superimposed in Fig. 5D. The arrows mark the synaptic potentials from the voltage trace recorded at t = 0 (black trace). Fig. 5E expands the region enclosed by the dotted box [Fig. 5B] to compare the AP driven by synaptic inputs (black and red trace; marked by arrows) with that generated spontaneously (blue trace). The ability to fire spontaneous AP is apparently aided by the unusually large peak AHP (∼**–**220 mV), in spite of relatively negative approximate RMP of **–**74 mV (dotted line), which allow for more depolarizing interspike potentials (marked by arrowheads) leading to APs.

### Peak AHP negative to calculated E_K_ may arise by membrane hyperpolarization

**Fig. 6:**
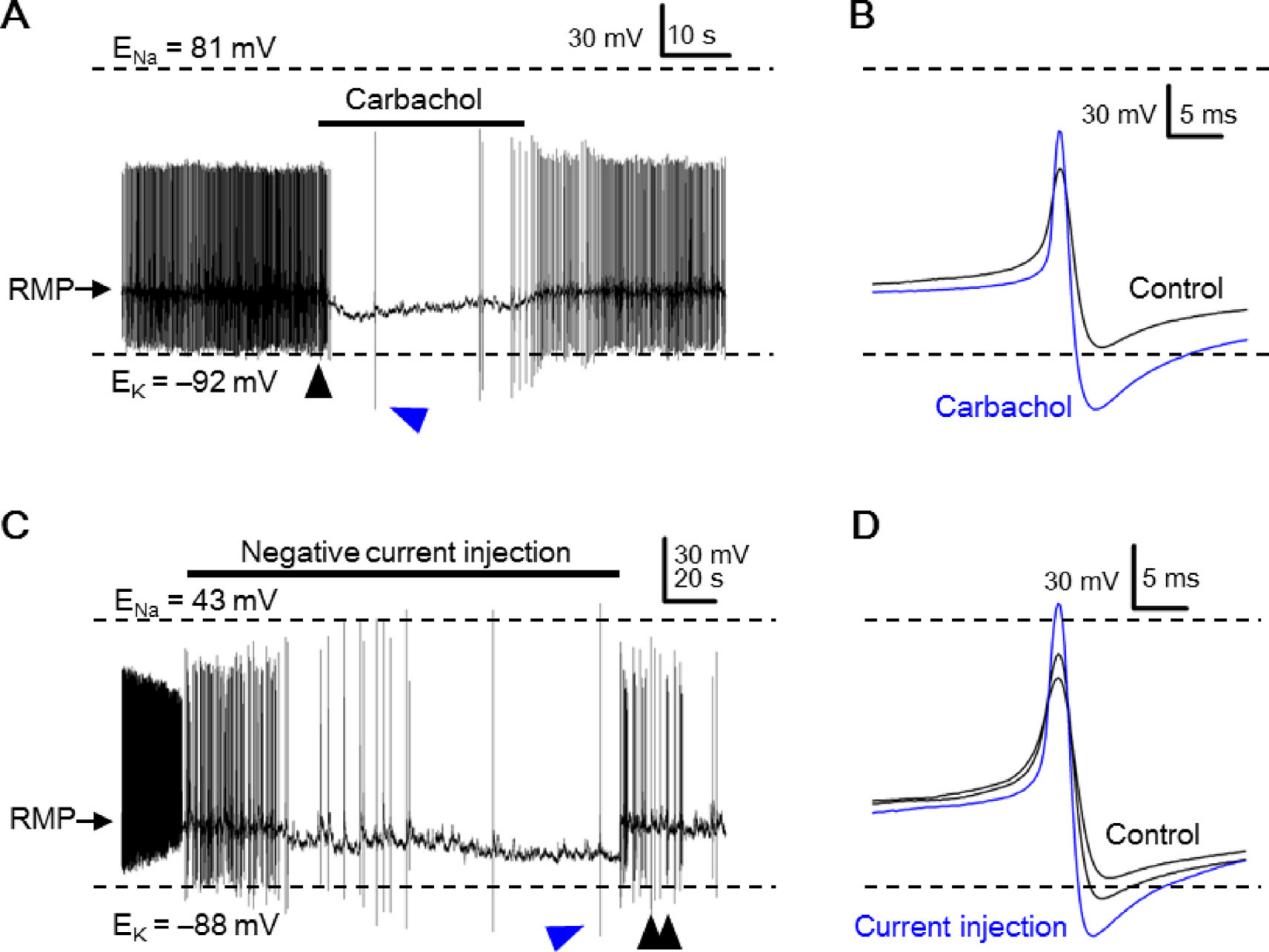
Reversible increase of peak AHP amplitude in response to hyperpolarizing stimuli. (A) A representative experiment to show the reversible effect of 100 μM carbachol on the spontaneous firing. Note the marked increase in the peak-to-peak amplitude of the remaining action potentials (APs) in carbachol. Arrowheads mark the two APs to be compared in (B). (B) Superimposition of APs to show the carbachol-induced increase in the peak AHP amplitude to become negative to calculated E_K_. (C) A representative experiment to show the reversible effect of negative current injection on the AP. Arrowheads mark the three APs to be compared in (D). (D) Superimposition of APs to show the negative current injection-induced increase in peak AP amplitude to become positive to calculated E_Na_ and peak AHP amplitude to become negative to calculated E_K_.

We have also encountered cells that increased peak AHP amplitude to become negative to calculated E_K_ in response to hyperpolarizing stimuli [Fig. 6]. We previously showed that carbachol, a nonspecific cholinergic agonist, mostly inhibits the SCN neurons by acting on muscarinic receptors to open at least background K^+^ channels, thereby hyperpolarizing RMP and suppressing spontaneous firing [28]. Fig. 6A shows the effect of 100 μM carbachol on the spontaneous firing in a representative SCN neuron. For this particular cell, cholinergic hyperpolarization markedly inhibited spontaneous firing and increased the peak-to-peak amplitude of the remaining APs to have peak AHP become negative to calculated E_K_. Fig. 6B compares the selected APs (marked by arrowheads, Fig. 6A) in control (black trace) and in carbachol (blue trace). For a total of 6 cells having their peak AHP increased by carbachol to become negative to calculated E_K_, the peak AHP amplitude changed from −82.8 ± 2.2 mV (n = 6) to −103.5 ± 5.9 mV (n = 6; *p* < 0.01; paired *t*-test) and the spontaneous firing rate from 4.5 ± 1.4 Hz (n = 6) to 0.3 ± 0.1 Hz (n = 6; *p* < 0.05; paired *t*-test).

Fig. 6C shows the first 6 min of voltage recordings from a cell after breaking into the whole cell condition. Negative current injection was applied shortly due to the gradual decrease in the peak-to-peak amplitude of APs, to even smaller than 80 mV, a condition signifying relatively depolarized, fluctuated RMP due to unhealthy or bad recording condition. As indicated, negative current injection reduced the rate of spontaneous firing and increased the amplitude of APs to the point that the peak AP became positive to calculated E_Na_ and the peak AHP negative to calculated E_K_. Termination of negative current injection rapidly depolarized the RMP and the APs became smaller again with fluctuated peak-to-peak amplitude. Fig. 6D compares the selected APs (marked by arrowheads, Fig. 6C) in the absence (black traces) and presence (blue trace) of negative current injection. Taken together, the rapid, reversible increase in peak AHP to become negative to calculated E_K_ suggests that local [K^+^]_o_ sensed by the AHP K^+^ channels is already lower than bulk [K^+^]_o_ but the AHP K^+^ conductance only become large (during hyperpolarizing stimuli) enough to generate peak AHP more negative than calculated E_K_.

### Ionic mechanisms for the peak AHP negative to calculated E_K_

**Fig. 7:**
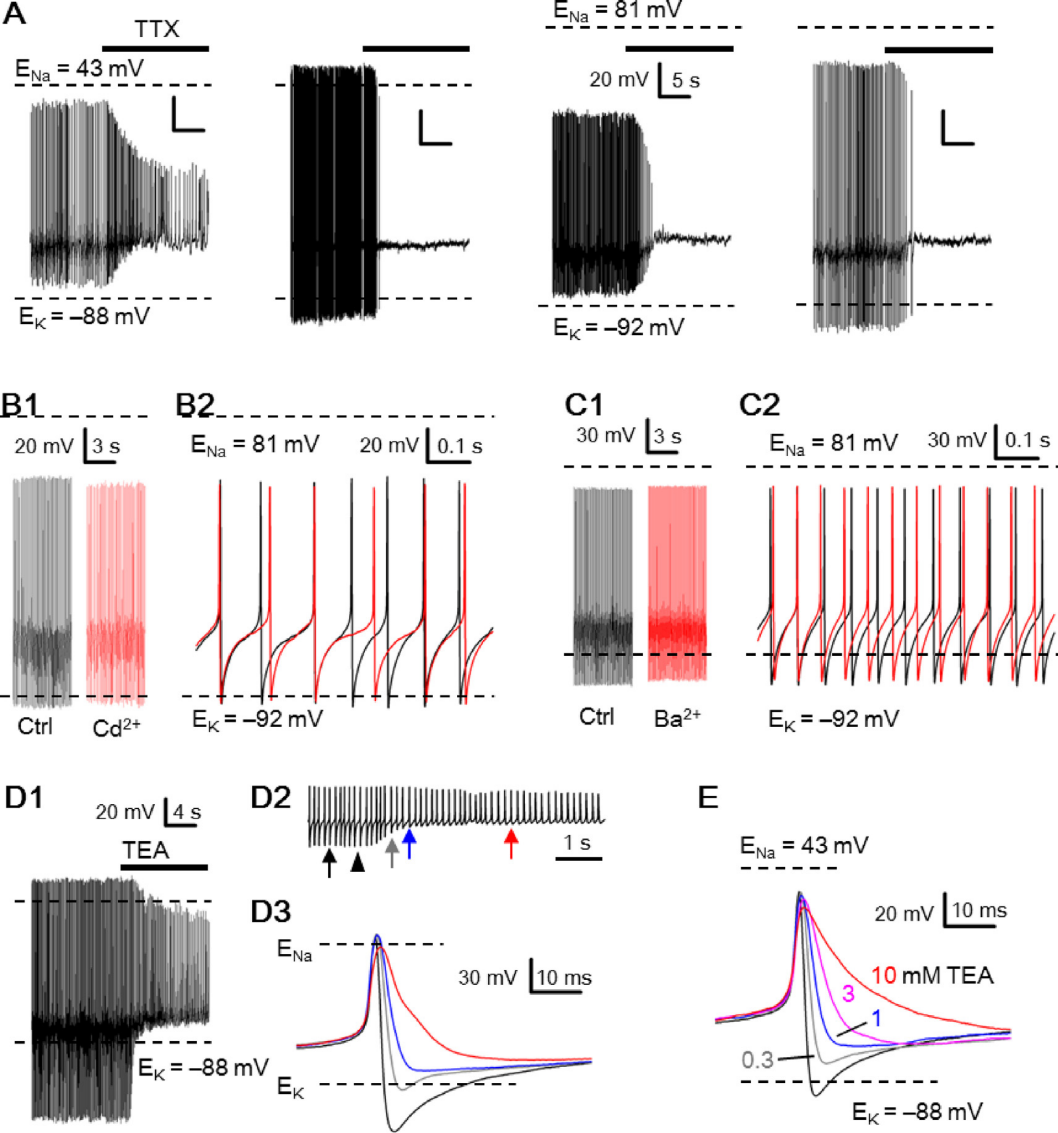
Effects of various channel blockers on the action potentials (APs). (A) Four representative SCN neurons to show the effect of 0.3 μM TTX on the spontaneous firing from cells recorded with high-Na^+^ (left two panels) and low-Na^+^ (right two panels) pipette solution. Note that TTX blocked APs of all but one (leftmost panel) cell. The scale labels in the middle right panel apply to all the rest of panels. (B1) A representative SCN neuron showing its spontaneous firing in control (left panel) and in the presence of 20 μM Cd^2+^ (right panel). (B2) Superimposition of APs in control (black trace) and in Cd^2+^ (red trace) for comparison. (C1) A representative SCN neuron showing its spontaneous firing in control (left panel) and in the presence of 3 mM Ba^2+^ (right panel). (C2) Superimposition of APs in control (black trace) and in Ba^2+^ (red trace) for comparison. (D1) A representative SCN neuron showing its firing response to 10 mM TEA. (D2) An epoch of 6-s recording (from (D1)) showing the first few seconds of response to TEA. Arrowhead marks the time when the TEA solution hits the cell. Arrows mark the APs to be compared in (D3). (D3) Superimposition of APs recorded before (black trace) and after TEA at different time points (grey, blue, and red trace). Note the rapid elimination of AHP by TEA. (E) A representative SCN neuron to show the dose-dependent effect of TEA (0.3, 1, 3, and 10 mM) on the AP. Note the marked reduction of peak AHP amplitude by 0.3 mM TEA.

Various channel blockers were used to determine the ionic mechanisms for APs with peak AHP negative to calculated E_K_, or even with peak AP positive to E_Na_ with high-Na^+^ pipette solution [Fig. 7]. Fig. 7A shows the effect of TTX on the spontaneous firing from cells recorded with high-Na^+^ (left two panels) and low-Na^+^ (right two panels) pipette solution. As expected, TTX at a concentration of 0.3 μM completely suppress the generation of APs in most cells (right three panels), leaving one cell firing Ca^2+^ spikes with much reduced peak AP and negligible AHP (leftmost panel). The results indicate that the TTX-sensitive Na ^+^ channels are responsible for the peak AP positive to calculated E_Na_ when recorded with high-Na^+^ pipette solution.

Fig. 7B–D show the effects of 20 μM Cd^2+^, 3 mM Ba^2+^, and 10 mM TEA on the APs, respectively, recorded from three representative cells. Although the peak AHP amplitude was reduced by Cd^2+^ and Ba^2+^, respectively, from −101 ± 2.1 mV (n = 4) to −97 ± 1.7 mV (n = 4; *p* < 0.01; paired *t*-test) and from −103 ± 5.4 mV (n = 4) to −99.5 ± 5.6 mV (n = 4; *p* < 0.05; paired *t*-test), it remained more negative than calculated E_K_. As 20 μM Cd^2+^ blocks most of the high-voltage-activated Ca^2+^ channels [29], and thus Ca^2+^-dependent K^+^ channels, and 3 mM Ba^2+^ blocks large-conductance Ca^2+^-dependent K^+^ channels (for review, see Ref. [30]), the results suggest a small contribution at best to the observed peak AHP negative to calculated E_K_. In contrast, 10 mM TEA markedly reduced the peak AHP amplitude [Fig. 7D1]. Fig. 7D2 shows an epoch of 6 s membrane voltages to indicate the time course of change in the peak AHP in response to the application of 10 mM TEA. Fig. 7D3 compares the selected APs (marked by arrows, Fig. 7D2) in control (black trace) and in the presence of 10 mM TEA (grey, blue, and red trace). As indicated, in ∼1 s into 10 mM TEA, the peak AHP (blue trace) has become positive to calculated E_K_. On average, the peak AHP amplitude was reduced by 10 mM TEA from −110.5 ± 5.3 mV (n = 8) to −72.5 ± 4.3 mV (n = 8; *p* < 0.01; paired *t*-test). Fig. 7E shows a different set of experiments to determine the dose-dependent effect of TEA on the AP. The result indicates that TEA at submillimolar concentrations effectively reduces the amplitude of peak AHP to become positive to calculated E_K_. The averaged peak AHP amplitude in control and in 0.3, 1, 3, and 10 mM TEA were, respectively, −96.2 ± 6.2 mV (n = 5), −73.6 ± 3.4 mV (n = 5), −66.4 ± 2.9 mV (n = 5), −60 ± 3 mV (n = 4), and −54 ± 2.3 mV (n = 4).

### Peak AHP negative to calculated E_K_ may also occur in perforated-patch recordings

**Fig. 8:**
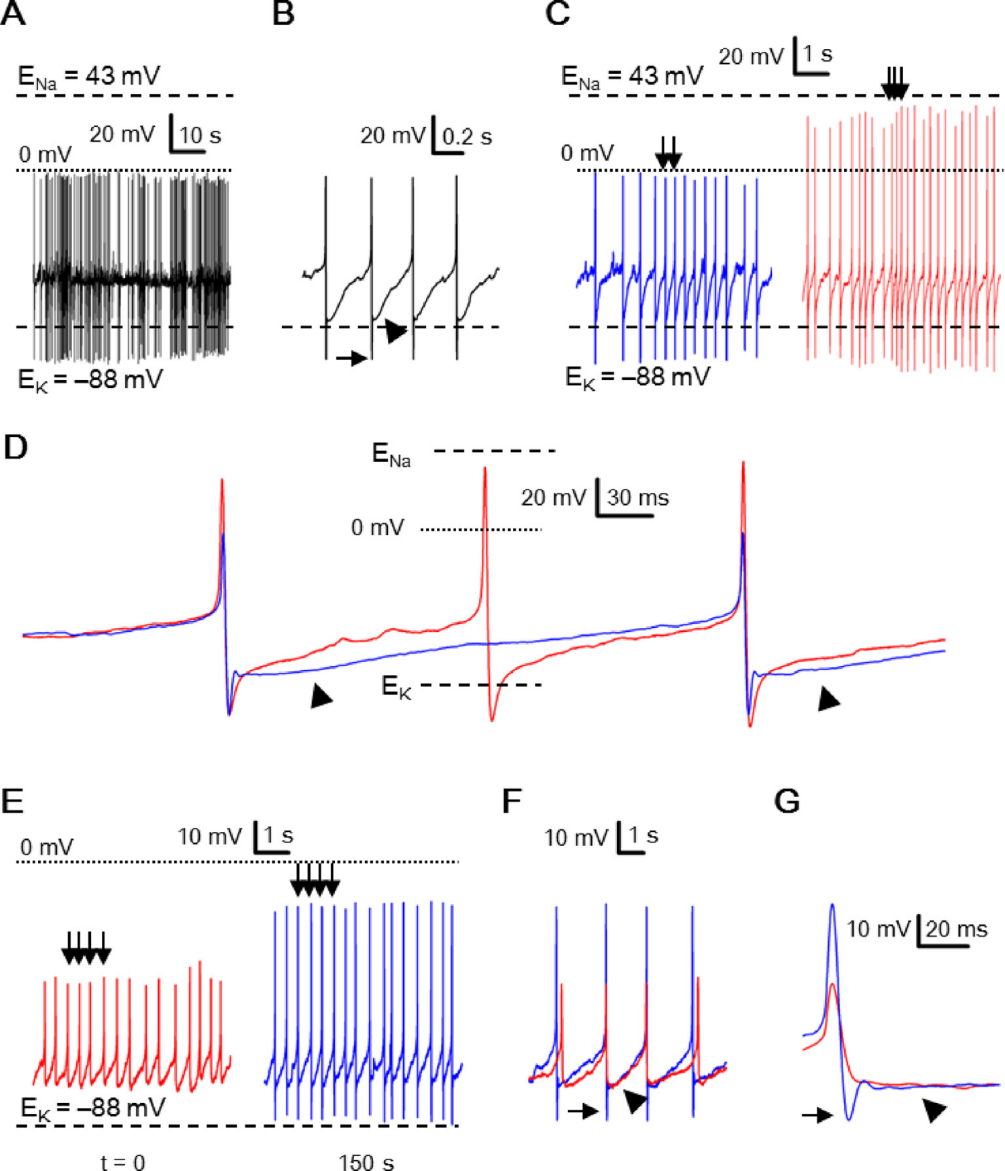
Perforated-patch recording of action potentials (APs). (A) A perforated-patch cell firing spontaneous APs with the peak AHP amplitude more negative than calculated E_K_. Note the small peak AP amplitude close to 0 mV. (B) A stretch of voltage trace (from (A)) to show the fast (marked by arrow) and slow (marked by arrowhead) AHP. (C) Comparison of APs recorded with perforated-patch (left panel; from (A)) and whole-cell (from the same cell shown in Fig. 4) recordings. Note the similar RMP and peak AHP, but much smaller peak AP in perforated-patch recordings. Arrows mark the APs to be compared in (D). (D) Comparison of perforated-patch and whole-cell APs indicates that the slow AHP (marked by arrowheads) prolongs the interspike interval. (E) A representative perforated-patch cell to show spontaneous firing recorded at t = 0 (left panel) and 150 s (right panel) after establishing stable recordings. Arrows mark the APs to be compared in (F). (F) Comparison of perforated-patch APs recorded at t = 0 and 150 s indicates a selective increase in the peak AP and peak fast AHP (marked by arrow), but not slow AHP (marked by arrowhead), at = 150 s, as better seen in the expanded time course shown in (G).

Although much less frequent than with whole-cell recordings, peak AHP negative to calculated E_K_ could also be observed in cells with perforated-patch recordings [Fig. 8]. For a total of 194 cells, all with high-Na^+^ pipette solution, 18 cells (9%) have peak AHP negative to calculated E_K_ (**–**88 mV), and none (0%) have peak AP positive to calculated E_Na_ (+43 mV). Fig. 8A shows the first minute of membrane voltage recorded from one such cell. Fig. 8B shows a selected stretch of recordings from Fig. 8A to indicate the presence of both fast AHP (marked by arrow) and slow AHP (marked by arrowhead) in perforated-patch APs. Note that only the peak amplitude of fast AHP, but not slow AHP, could become negative to calculated E_K_. Compared to the whole-cell APs in cells recorded with also high-Na^+^ pipette solution, two features could be readily distinguished. First, the peak AP amplitude is always small for perforated-patch APs, close to 0 mV at best, irrespective of the peak AHP amplitude. The much smaller peak AP amplitude is most likely due to the high access resistance of perforated-patch recording configuration created by the use of pore-forming nystatin, which would preferentially reduce the amplitude of fast changing voltage as in the upstroke of APs.

Second, as indicated in Fig. 8B, the perforated-patch AP has slow AHP (marked by arrowhead) in addition to fast AHP (marked by arrow) (see also Fig. 8D). To better illustrate this point, we compare the APs obtained with perforated-patch and whole-cell recordings [Fig. 8C and D]. Fair comparison is ensured by selecting the whole-cell voltage having similar RMP and peak AHP to match with the perforated-patch voltage as shown in Fig. 8C. The 6-s epoch of whole-cell voltage (right panel) is from the same cell shown in Fig. 4 at ∼5 min after breaking into the whole-cell configuration. Note that the whole-cell APs (right panel; 4.7 Hz) have much larger peak AP amplitude albeit with similar RMP and peak AHP as the perforated-patch APs (left panel; 2.2 Hz). Fig. 8D superimposes the selected APs (marked by arrowheads, Fig. 8C) recorded with whole-cell (red trace) and perforated-patch (blue trace) configuration, indicating that the presence of slow AHP (marked by arrowheads) delays the depolarization of the interspike potential and likely contributes to the lower firing rate in perforated-patch recordings.

In some experiments when nystatin-mediated membrane perforation was slow to allow for clear visualization of the changes in the AP waveform, it may provide an opportunity to determine the dependence of recorded voltage on membrane perforation. Fig. 8E shows two 6-s epochs of membrane voltage recorded at t = 0 (left panel) and 150 s (right panel) into stable perforated-patch recordings. Fig. 8F compares the selected APs (marked by arrows, Fig. 8E) recorded at t = 0 (red trace) and at t = 150 s (blue trace). The result indicate that as time passed the peak AP become larger along with the emergence of fast AHP (marked by arrow), whereas the slow voltage change such as RMP and slow AHP (marked by arrowhead) were virtually unaltered. Fig. 8G expands the time course to better visualize the preferential increase in the amplitude of fast changing voltage as membrane perforation improves.

## Discussion

The SCN is tightly packed with small neurons and has abundant perikaryal appositions which may allow for non-synaptic communications such as ephaptic and ionic interactions. In particular, the closely apposed cell bodies with morphological specializations in the narrow ECS [16] could slow molecular diffusion to permit ion accumulation or depletion in the diffusion-restricted ECS. In this study, we show that the SCN neurons can fire APs with TEA-sensitive peak AHP amplitude more negative than calculated E_K_, indicating that local [K^+^]_o_ in the ECS sensed by the TEA-sensitive AHP K^+^ channels must be lower than bulk [K^+^]_o_. This may occur if NKA is also present in the membrane regions facing the diffusion-restricted ECS, so as to control local [K^+^]_o_ to exert specific regulation of peak AHP amplitude and thus neuronal excitability.

### Action potentials (APs) with peak AHP more negative than calculated E_K_

We show that the rat SCN neurons could fire spontaneous APs with the amplitude of peak AHP more negative than calculated E_K_ irrespective of high-Na^+^ [Fig. 1] or low-Na^+^ [Fig. 2] pipette solution. The TEA-sensitive peak AHP amplitude more negative than calculated E_K_ could occur only if local [K^+^]_o_ in the ECS sensed by the TEA-sensitive K^+^ channels is lower than bulk [K^+^]_o_ (3.5 mM in bath solution) or submembrane local [K^+^]_i_ sensed by the TEA-sensitive K^+^ channels higher than bulk [K^+^]_i_ (110 or 130 mM in pipette solution). Given a recorded value of peak AHP = **–**110 mV, for example, a simple calculation according to the Nernst equation indicates that local [K^+^]_o_ in the ECS should be lower than 1.5 (1.8) mM with 110 (130) mM K^+^ pipette solution or submembrane local [K^+^]_i_ higher than 254 mM, the latter of which is not attainable due to osmotic pressure. Thus to achieve a peak AHP of **–**110 mV, the energized NKA must transport K^+^ against its electrochemical gradient to maintain local [K^+^]_o_ in the ECS lower than 1.5 (1.8) mM (compared to bulk [K^+^]_o_ of 3.5 mM) to be sensed by the TEA-sensitive AHP K^+^ channels. In other words, both NKA and TEA-sensitive AHP K^+^ channels should be localized to the membrane regions facing the K^+^ diffusion-restricted ECS.

Nevertheless, cells with local [K^+^]_o_ in the ECS lower than bulk [K^+^]_o_ will not guarantee to fire APs with peak AHP amplitude large enough to become negative to calculated E_K_. This is because the peak AHP amplitude depends on both the driving force and the K^+^ conductance. As such, cells with lower local [K^+^]_o_ (i.e. larger driving force) but small K^+^ conductance could not have large enough peak AHP to become negative to calculated E_K_. This is best illustrated by the observation of hyperpolarization-evoked rapid, reversible increase in peak AHP to become negative to calculated E_K_ [Fig. 6]. The concomitant increase in both peak AP and peak AHP in response to hyperpolarizing stimuli suggests that membrane hyperpolarization removes resting inactivation of TTX-sensitive Na^+^ channels (and other voltage-dependent inward current) to increase peak AP, which in turn increase K^+^ conductance and driving force as well to markedly increase the peak AHP to go beyond calculated E_K_. In this context, it is not entirely impossible that every SCN neurons have the potential to fire APs with peak AHP large enough to go beyond calculated E_K_.

The TEA-sensitive AHP is most likely mediated by the fast delayed rectifier K^+^ channels Kv3.2 and Kv3.1b, which are present in most cell bodies in the mouse SCN [31,32]. These channels are activated at more positive voltages and deactivate very rapidly to allow for high-frequency repetitive firing in many central neurons [33,34]. In particular, Kv3.1b proteins are predominantly expressed in the somatodendritic membrane in the mouse brain [35]. In cerebellar granule cells there are non-uniform distributions of Kv3.1b on the somata, with circular bands of labeling near the axon hillock [36], and in cultural hippocampal neuron, the adaptor protein Ankyrin-G targets Kv3.1b to the axon hillock [37]. Ankyrin-G also colocalizes with Kv3.1b and with Na_V_1.1 channel at axonal nodes and heminodes [38,39]. Most recently, Ankyrin-R is found to directly interact with Kv3.1b and is both necessary and sufficient for clustering Kv3.1b K^+^ channels in the soma to regulate excitability of GABAergic interneurons [40]. RT-PCR analysis showed that the rat SCN expresses mRNAs for all three ankyrin isoforms, Ankyrin-R, Ankyrin-B, and Ankyrin-G (Wan and Huang, unpublished observation). Further work is warranted to investigate the somatic colocalization of Kv3.1b with NKA α-isoforms and with Ankyrin-G or Ankyrin-R, in a hope to help localize the presumed K^+^ diffusion-restricted ECS regions in the rat SCN.

### Action potentials (APs) with peak AP more positive than calculated E_Na_

The SCN neurons could also fire spontaneous APs with peak AP more positive than calculated E_Na_ if pipette was filled with high-Na^+^ solution. The peak AP amplitude more positive than calculated E_Na_ (+43 mV) could occur only if local [Na^+^]_o_ in the ECS sensed by the TTX-sensitive Na^+^ channels is higher than bulk [Na^+^]_o_ (140 mM in bath solution) or submembrane local [Na^+^]_i_ sensed by the TTX-sensitive Na^+^ channels lower than bulk [Na^+^]_i_ (26 mM in pipette solution). Given a recorded value of peak AP = +60 mV, for example, local [Na^+^]_o_ in the ECS sensed by the TTX-sensitive Na^+^ channels should be higher than 269 mM or submembrane local [Na^+^]_i_ lower than 16 mM, the former of which is not attainable due to osmotic pressure. Thus to achieve a peak AP of +60 mV, the energized NKA must transport Na^+^ against its electrochemical gradient to maintain submembrane local [Na^+^]_i_ to lower than 16 mM (compared to bulk [Na^+^]_i_ of 26 mM) to be sensed by the TTX-sensitive Na^+^ channels.

Further support for submembrane local [Na^+^]_i_ being different from bulk [Na^+^]_i_ comes from the observation that cells recorded with high-Na^+^ (26 mM Na^+^) or low-Na^+^ (6 mM Na^+^) pipette solution had similar peak AP amplitude despite the marked difference in calculated E_Na_ between high-Na^+^ (E_Na_ = +43 mV) and low-Na^+^ (E_Na_ = +81 mV) [Fig. 3A and B]. A simple explanation for this is that both have similar submembrane local [Na^+^]_i_ around the TTX-sensitive Na^+^ channels, although their bulk [Na^+^]_i_ differ markedly, being 26 mM and 6 mM. To estimate submembrane local [Na^+^]_i_ sensed by the TTX-sensitive Na^+^ channels, we considered only large peak AP amplitude, for example, between +55 mV and the largest recorded amplitude of +67.5 mV, so that the recorded peak AP would be close to and thus a good measure of true E_Na_. A simple calculation according to the Nernst equation yields 15 mM and 10 mM submembrane local [Na^+^]_i_, respectively, for peak AP amplitude of +55 mV and +67.5 mV, assuming [Na^+^]_o_ = 140 mM clamped by the bath solution. The assumption of [Na^+^]_o_ being clamped at 140 mM by the bath solution is reasonable even if the TTX-sensitive Na^+^ channels also reside in the K^+^ diffusion-restricted ECS, because [Na^+^]_o_ is unlikely to increase too much beyond 140 mM due to the constrain imposed by the corresponding increase in the osmotic pressure.

It is interesting to note that the largest peak AP amplitude recorded with either high-Na^+^ or low-Na^+^ pipette solution was incidentally the same, with a value of +67.5 mV. This may set a lower limit concentration of ∼10 mM submembrane local [Na^+^]_i_ around the TTX-sensitive Na^+^ channels. No matter what submembrane local [Na^+^]_i_ is, it is a result of combined actions of NKA-mediated Na^+^ extrusion and Na^+^ entry via the TTX-sensitive Na^+^ channels and non-desensitizing Na^+^ conductances. In this context, it is worth mentioning of our previous findings suggesting that submembrane local [Na^+^]_i_ around the NKA Na ^+^ pump sites is not clamped by the pipette solution [27]. Specifically we demonstrated finite NKA Na^+^ pumping activity with Na^+^-free pipette solution in the presence of TTX, suggesting finite submembrane local [Na^+^]_i_ around the NKA Na ^+^ pump sites, apparently mediated by Na^+^ entry via TTX-insensitive Na^+^ routes [27]. Nevertheless, there remains a possibility that the pipette solution may not sufficiently diffuse into the dendrites during the recording period and only partially modifies [Na^+^]_i_.

### Comparison of whole-cell and perforated-patch APs

The SCN neurons could also, but rarely, fire APs with peak AHP more negative than calculated E_K_ with perforated-patch recordings. The rare occurrence of peak AHP negative to calculated E_K_ is most likely due to the less than perfect electrical access between the recording electrode and cytosol for perforated-patch compared to whole-cell recordings. The high access (series) resistance would low-pass filter the measured signal, thereby attenuating particularly the fast changing voltage such as the upstroke of an AP, as suggested by comparing APs recorded with perforated-patch and whole-cell recordings [Fig. 8C and D]. The preferential attenuation of fast changing voltage with perforated-patch recordings could also be demonstrated by the result of selective increase in both the peak AP and peak AHP as time passes to achieve better membrane perforation by nystatin [Fig. 8E–G]. The same reasoning can also explain the lack of observation of peak AP positive to calculated E_Na_ with high-Na^+^ pipette solution.

An additional difference is the presence of slow AHP with perforated-patch, but not whole-cell, recordings [Fig. 8D]. This difference is most likely due to the buffering of cytosolic Ca^2+^ by EGTA in the pipette with whole-cell recordings. Indeed, our preliminary result indicated that the slow AHP is mediated by Ca^2+^-dependent K^+^ current. The presence of slow AHP, as opposed to fast AHP, prolongs the interspike interval and may contribute to the lower spontaneous firing rate recorded with cell-attached and perforated-patch recordings than with whole-cell recordings (unpublished results).

### K^+^ diffusion-restricted extracellular space (ECS) in the rat SCN

The results, peak AHP amplitude more negative than calculated E_K_, presented in this study indicates that local [K^+^]_o_ in the ECS sensed by the TEA-sensitive K^+^ channels must be lower than bulk [K^+^]_o_. To account for this, note that local [K^+^]_o_ in the narrow ECS can be described with a macroscopic diffusion equation that includes terms for diffusion, source (K^+^ release via K^+^ channels), and sink (K^+^ clearance via NKA) [41]. The diffusion of K^+^ is hindered due to the narrow ECS with intricate structure that effectively slows the diffusion of K^+^ compared to unrestricted free diffusion in bulk solution. When aided by K^+^ clearance via NKA in the small volume of ECS, local [K^+^]_o_ around the TEA-sensitive AHP K^+^ channels could become lower than bulk [K^+^]_o_.

The suggested presence of K^+^ diffusion-restricted ECS is not inconsistent with the ECS geometry in the rat SCN. The SCN is tightly packed with small size of neurons, which are among the smallest in the brain, as well as abundant perikaryal appositions [16]. The neuronal cell bodies are closely apposed with or without intervening glial elements, and closely apposed neurons without an intervening glial element may possess electron-dense materials or punctate densities between them. There are also unusual series of punctate junctions, so-called zipper junctions, between cell bodies and sometime between cell body and axon terminals. Interestingly, many mitochondria, along with smooth endoplasmic reticular, are seen close to the zipper junction between cell bodies. Close appositions between neurons and glia can also be revealed by electron microscopic analysis [16]. It is likely that the abundant perikaryal appositions with morphological specializations in the narrow ECS increases the tortuosity to slow the diffusion of K^+^, allowing it to be cleared by NKA to maintain a lower local [K^+^]_o_ in the ECS than bulk [K^+^]_o_.

### Functional implications

The brain ECS is composed of fluid-filled spaces laden with extracellular matrix and measurement of ECS tortuosity suggests that the ECS is a highly connected space so that any location in the ECS could be reached by multiple pathways except for some dead-space microdomains [[42], [43], [44]]. In essence, the ECS supplies a reservoir of ions for electrical signalling and mediates chemical signalling through volume transmission [43,44]. Nevertheless, local K^+^ fluctuation is not considered very useful as a signalling molecule due to its nonspecific effect on membrane potentials [43,44]. Our results suggest that this may not be the case in the rat SCN. We showed that only TEA-sensitive fast AHP, but not RMP, could become negative to calculated E_K_. Furthermore, no change in RMP was observed during the course of spontaneous increase in peak AHP to become negative to calculated E_K_ [Fig. 4]. The results suggest differential localization of K^+^ channels for AHP and for RMP to different membrane regions facing different local environments. Most likely, both the TEA-sensitive AHP K^+^ channels and NKA are at membrane regions facing the same K^+^ diffusion-restricted ECS such that NKA can clear K^+^ to maintain a lower local [K^+^]_o_ than bulk [K^+^]_o_. In the case of TEA-sensitive AHP K^+^ channels, local [K^+^]_o_ may be controlled to specifically regulate the peak AHP. Different combinations of NKA and other K^+^ channels in other similarly K^+^ diffusion-restricted ECS may regulate local [K^+^]_o_ to play different, specific roles. In this sense, local K^+^ fluctuation could be useful as a signalling molecule in K^+^ diffusion-restricted ECS.

The functional presence of K^+^ diffusion-restricted ECS, in which local [K^+^]_o_ is balanced by K^+^ clearance via NKA and K^+^ release via K^+^ channels, may allow for K^+^-mediated ionic interactions among neurons to regulate SCN physiology. Our results suggest such a possibility at least for the AHP, which plays a role in the regulation of AP firing in the SCN neurons [[45], [46], [47], [48], [49], [50]]. Our results show that, at least with whole-cell recordings, cells having larger peak AHP, particularly when negative to calculated E_K_, had higher firing rate. In other words, local [K^+^]_o_ in the ECS around and sensed by the TEA-sensitive AHP K^+^ channels plays a role in the regulation of neuronal excitability. Intercellular coupling via ionic interactions among neurons may become possible if the K^+^ diffusion-restricted ECS is shared locally, or even more broadly, by a number of cells.

## Conclusion

The rat SCN neurons can fire APs with TEA-sensitive peak AHP more negative than calculated E_K_. The result indicates that local [K^+^]_o_ in the ECS around and sensed by the TEA-sensitive K^+^ channels must be lower than bulk [K^+^]_o_. Such a lower local [K^+^]_o_ can be achieved by NKA-mediated K^+^ clearance in K^+^ diffusion-restricted ECS. Since local [K^+^]_o_ in the K^+^ diffusion-restricted ECS regulates peak AHP, which plays a role in regulating SCN neuronal firing, the K^+^ diffusion-restricted ECS may allow for K^+^-mediated ionic interactions among neurons to regulate SCN excitability.

## Funding

This work was supported by 10.13039/501100004606Chang Gung Medical Foundation (CMRPD1K0691; R.C.H) and by Taiwan 10.13039/501100003711Ministry of Science and Technology (MOST108-2320-B-182-017-MY3; R.C.H).

## Conflicts of interest

None.
